# Production Protocol Standardisation, Macroscopic and Histological Evaluation, and Growth Factor Quantification of Canine Leukocyte-and Platelet-Rich Fibrin Membranes

**DOI:** 10.3389/fvets.2022.861255

**Published:** 2022-04-15

**Authors:** Chiara Caterino, Giovanni Della Valle, Federica Aragosa, Davide De Biase, Gianmarco Ferrara, Francesco Lamagna, Gerardo Fatone

**Affiliations:** ^1^Department of Veterinary Medicine and Animal Production, University of Naples “Federico II”, Naples, Italy; ^2^Department of Pharmacy/DIFARMA, University of Salerno, Fisciano, Italy

**Keywords:** platelet concentrates, dog, bioscaffold, growth factors, ELISA, regenerative medicine, wound, fibrin mesh

## Abstract

Leukocyte-Platelet-Rich Fibrin (L-PRF) is a second generation of platelet concentrates; it was widely used, as an autologous platelet-based wound sealant and hemostatic agent in surgical wound healing. L-PRF clot or membrane is a solid fibrin-based biomaterial, with a specific 3D distribution of the leukocytes and platelet aggregates. This biological scaffold releases growth factors (i.e., TGF- β1, PDGF-AB, VEGF) and matrix proteins (fibronectin, vitronectin and thrombospondin-1) during the healing process after the application. To the Authors' knowledge both in human and veterinary medicine a single standardised protocol was not reported. This prospective study aimed to apply Crisci's L-PRF protocol (which is characterised by 30” of acceleration, 2' at 2,700 rpm, 4' at 2,400 rpm, 3' at 3,000 rpm, and 36” of deceleration and arrest) sin canine species, evaluate macroscopically and histologically the L-PRF membranes obtained by using Wound Box to standardise the L-PRF protocol in dogs and to evaluate the clinical feasibility of using L-PRF membranes by quantitative *in vitro* analysis of growth factors over 7 days. One hundred twenty-eight dogs in good general condition with no history of recent NSAIDs intake (15 days of washout) and/or any medication or disease related to coagulation process met inclusion criteria and therefore were enrolled. We obtained 172 membrane L-PRF membranes by 86 dogs: half of them underwent macroscopic and histological analysis, the other 86 underwent ELISA analysis. The Wound Box gave a membrane of mean (±SD) length (cm), width (cm) and weight (g) of 1.97 (±0.89), 0.95 (±0.36), 0.46 (±0.20) respectively. Histology analysis confirmed a well-defined histoarchitecture with five layers reproducing density and distribution of blood cells in this biomaterial. Finally, the ELISA assay performed with 22 L-PRF membranes showed a peak in growth factors at 6 h after membrane production, followed by a decrease in release at 24 and 72 h and a second peak in release at 168 h after production. Statistical analysis of demographic variables (age, sex, and body condition score BCS) and the average of growth factors determined by the ELISA assay did not reveal statistical significance, except for the BCS factor compared with the production of VEGF. Our data confirm the effectiveness of this protocol and of Wound Box to produce L-PRF membranes in dogs.

## Introduction

Regenerative medicine and tissue engineering have gained increasing interest in human and veterinary medicine. Platelet concentrates are autologous blood products that have become widely used since their introduction in dental and -maxillofacial surgery (1), sports medicine, and orthopaedics (2, 3). Dohan Ehrenfest et al. (4) classified platelet concentrates into the following four main families based on the presence of leukocytes and fibrin architecture: pure platelet-rich plasma, which does not contain leukocytes and forms a low-density fibrin network after activation; leukocyte- and platelet-rich plasma, which contains leukocytes and forms a low-density fibrin network; pure platelet-rich fibrin, which does not contain leukocytes and forms a high-density fibrin network; and leukocyte- and platelet-rich fibrin (L-PRF), which contains leukocytes and forms a high-density fibrin network (5). L-PRF is a second-generation platelet-derivate bioscaffold obtained by a whole physiological process and is characterised by the high concentration of platelets, which can release growth factors (GFs), such as platelet-derived growth factor (PDGF-AA, PDGF-AB, PDGF-BB), transforming growth factor-β1 (TGF-β1), vascular endothelial growth factor (VEGF), and other important proteins in the surrounding tissue (3). The effect of platelet-rich fibrin (PRF) on the cell behaviour of fibroblasts involved in soft tissue wound healing, endothelial cells, and GF release from various PRF formulations has been investigated in humans *in vitro* and *in vivo* (6). The results showed that PRF induces the proliferation of dermal fibroblasts, gingival fibroblasts, and keratinocytes, and that it participates in the production of extracellular matrix collagen type 1 (3). Moreover, soft and hard tissue healing and inflammation control are improved by GF release when platelets are entrapped in a fibrin membrane (7, 8). In veterinary literature, the protocols that describe the use of PRF refer mainly to dogs as a model for human medicine. Furthermore, inconsistent centrifugation protocols and centrifugation force units prevent objective comparison and evaluation of L-PRF use in dogs. In most cases, these protocols involved centrifugation at 3,000 rpm for 10 min (9–13), 1,300 rpm for 8 min (14), or 2,700 rpm for 12 min (15), and some protocols used 200 *g* to 400 *g* for 8 to 10 min (16–19). In 2001, Choukroun et al. reported a PRF production protocol for accumulating platelets and releasing cytokines in a fibrin clot by rapid human blood collection and centrifugation (1). Subsequently, other centrifugation protocols were reported, which vary from 400 *g* for 10 or 12 min (20–22) to 3,000 rpm for 10 min (12), or 1,100 *g* for 6 min, followed by 4,500 *g* for 25 min (23). In 2017, Crisci et al. (24) proposed a slight modification of the protocol developed by Choukroun et al. and standardised the production of L-PRF in horses for application to human production. In 2021, Soares et al. (10) proposed a protocol for producing PRF clots by centrifuging the venous blood of cats and dogs, and they investigated the biological properties and release profile of GFs and other cytokines. However, the likelihood of L-PRF clot and membrane formation by specific devices as well as the macroscopic and histological evaluation and the assessment of GF release has not been investigated in dogs.

The primary aim of this prospective study was to standardise the production of clots in dogs using the protocol described by Choukroun et al. (1) and modified by Crisci et al. (24), and we also intended to standardise the L-PRF membrane production using a device called the L-PRF Wound Box. The secondary aim was to evaluate the clinical feasibility of using L-PRF membranes by quantitative *in vitro* analysis of GFs over 7 days. We performed macroscopic evaluation, histological analysis, and GF quantification of the L-PRF membranes. Our first hypothesis was that the protocol is suitable for producing L-PRF clots in dogs and our second was that the compression of clots with the L-PRF Wound Box creates membranes macroscopically and histologically suitable for clinical use.

## Materials and Methods

For this prospective study, the protocols and procedures were reviewed and approved by the Ethical Animal Care and Use Committee of the University of Naples “Federico II” (prot. No. PG/2018/0113033 of 28/11/2018).

The study population consisted of 300 dogs housed in a private shelter in Caserta, Campania, Italy. All dogs routinely undergo a clinical examination, complete blood count (CBC), serum biochemistry, and IFAT test (Indirect immunofluorescent antibody test) for the diagnosis of canine leishmaniasis and ehrlichiosis. During the routine clinical examination, between September and October 2020, a whole blood sample of 20 mL was taken and divided into three portions of 1 mL for CBC, 1 mL for biochemistry and IFAT test, and an 18 mL portion, which was divided further into two samples for L-PRF clot and membrane production. Of the two membranes obtained, one was used for macroscopic and histological evaluation, whereas the other was used for quantifying GFs.

Exclusion criteria were presence of previous or concomitant diseases characterised by thrombocytopenia or platelet loss; concomitant pregnancy status; neoplasm, infectious disease, or septic status; history of recent NSAIDs or corticosteroids intake (15 days of minimum washout was accepted); any medication or disease related to coagulation; age <12 months; body weight <20 kg; body condition score <3 (scale of 1–5); or any alteration in CBC values.

Patients from whom we were able to obtain only one membrane from two blood samples were also excluded because they would not have allowed simultaneous histological and ELISA analysis.

If the dogs had not met the inclusion criteria, they would have been excluded *a priori* according to the anamnesis and clinical history provided by veterinary staff of the shelter. Furthermore, they would have their blood collected not for the study purpose, but for the routine clinical examination.

Data recorded included the formation of L-PRF clots after centrifugation; formation of L-PRF membranes after compression; length, width, and weight of membranes; histological analysis; and GF quantification by ELISA.

### Sample Collection

Before sampling, all dogs were allowed to explore freely in the consultation room for at least 15 min to familiarise themselves with the environment and the operators. After routine clinical examination, 18 mL of blood was quickly harvested by jugular venous sampling using a vacutainer (BD Vacutainer Safety-Lok-Blood Collection Set 21 g × 3/4” × 7”, Becton, Dickinson and Company), divided into two 9 mL samples for L-PRF clot production, and stored in two vacutainers (BD Vacutainer, Becton, Dickinson and Company).

### Centrifugation Protocol and Preparation of L-PRF Membranes

Within 2 min of sampling, the tube was centrifuged using a desk centrifuge with a radius of rotor of 100 mm (TD4A-WS, In LoveArts) according to the following protocol (24), which consisted of 30 s acceleration, 2 min at 2,700 rpm (816 g), 4 min at 2,400 rpm (645 g), 3 min at 3,000 rpm (1,008 g), and 36 s deceleration and stopping (Table 1). Room temperature was 25°C, the temperature inside the centrifuge was measured by a thermometer (TEMP 7 NTC, XS Instruments) with a probe (NT 7L, XS Instruments).

**Table 1:** Centrifugation protocol.

**Time**	**Centrifuge speed (rpm)**
30 s	Acceleration
2 min	2,700
4 min	2,400
3 min	3,000
36 s	Deceleration

**Figure 1:**
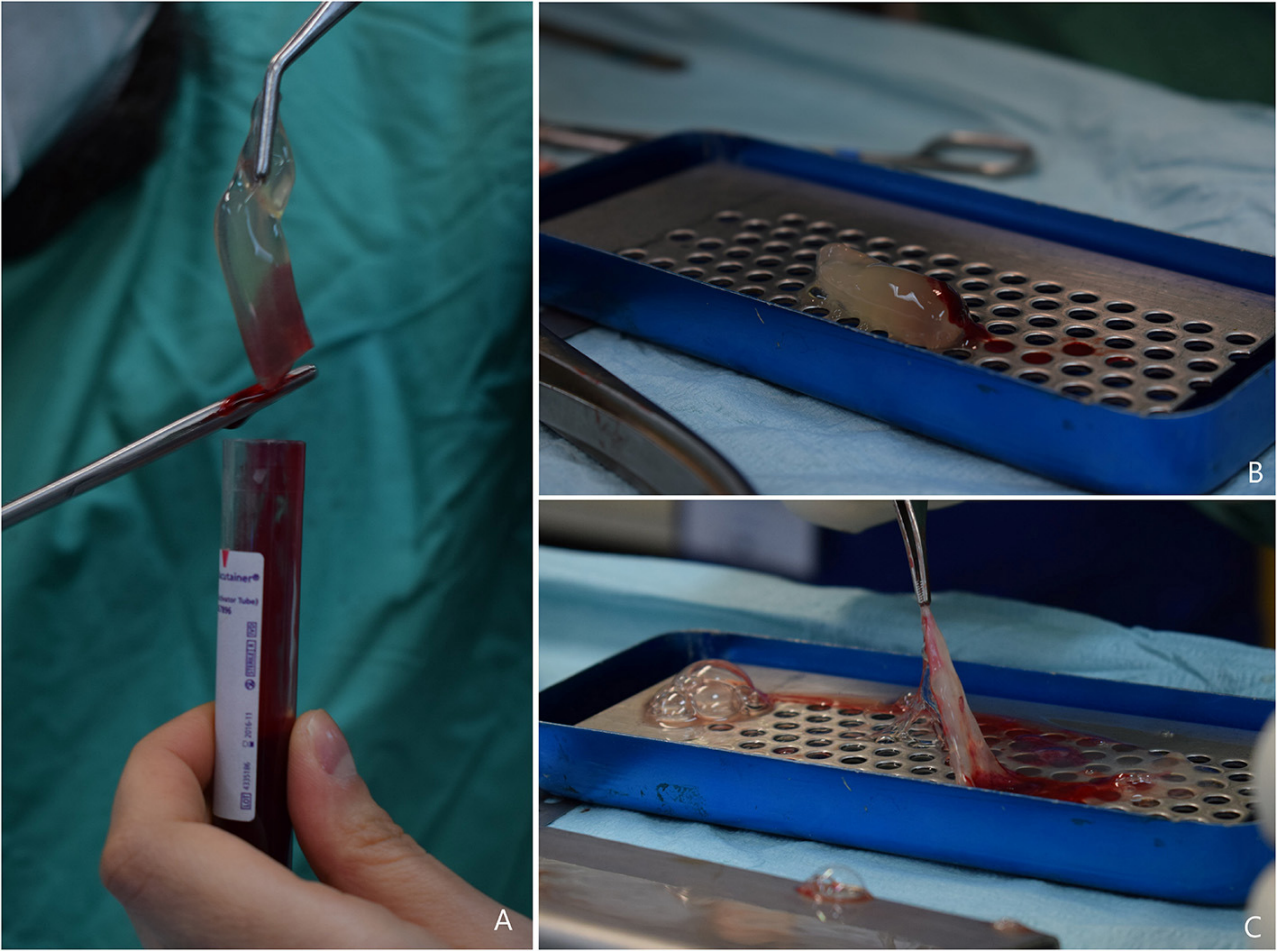
**(A)** L-PRF clot obtained after centrifugation, the red clot is gently removed using scissors; **(B)** L-PRF inside the Wound Box (*courtesy of Dr. A. Crisci*); **(C)** L-PRF membrane obtained after 15 min compression.

At the end of centrifugation, three layers were visible in the tube: the platelet-free plasma on the top, the fibrin clot (L-PRF clot) in the middle, and the red clot, consisting of red blood cells, at the bottom. The red clot was gently removed using a pair of scissors and the fibrin clot was placed in the L-PRF Wound Box (Figure 1). The wound box is made by using a metal container 17.5 × 7.6 × 2 cm containing a perforated steel plate of 150 × 68 × 1.5 mm. There was a second steel plate which acts as a compressor, 150 × 68 × 1.5 mm, with a weight of 148 g. This second shaped plate exerts a pressure of 142.437 Pa/cm^2^. Inside the L-PRF Wound Box, the steel plate compressor provided a homogeneous compression for 15 min.

### Macroscopic Analysis and Histological Analysis

Immediately after formation, the width and length (centimetres) of each L-PRF membrane was measured using a precision calliper (CDJB15-LTF, Borletti), and they were weighed (grammes) using a goldsmith's scale (PLC200B-C, G&G). The membranes were then stored in two sterile tubes (Demas, tubes in PS, 3 ml, 12 × 55 mm, cod. 00579192) and transported at 4°C to the laboratory within 6 h for morphological assessment.

The membranes collected for histological analysis were preserved in 10% neutral buffered formalin (code no. 05-01007Q, Bio-Optica) and stored at room temperature. Samples were subsequently dehydrated through graded alcohols before being embedded in paraffin wax. Sections 5-μm-thick were cut and stained with hematoxylin and eosin for morphological analysis. A semi-quantitative evaluation was also conducted for each section by two independent pathologists with a concordance rate of 95%. The scoring was adapted from Hamed and Hasouni (25) as follows:

**Zone layers in the membrane**: the demarcation between membrane layers was described as well (score 1) or poorly demarcated (score 2).

**Number of cell layers at border:** the number of cell layers at the periphery of clot were counted as more than ten layers (score 1) or less than ten layers (score 2).

**Aggregation of cells in the cell layer zone:** the proximity of cells to each other was considered as either heavy (score 1) or light (score 2).

**Cell border morphology:** cell border morphology was evaluated as either very clear (score 1), clear (score 2), very unclear (score 3).

### GF Quantification

Finally, membranes from 22 dogs were randomly selected for growth factor analysis by ELISA assay. The membranes collected for ELISA were placed in a 10 mL tube with 4 mL of sterile Dulbecco's Modified Eagle's Medium (DMEM; 11965092, Gibco). Then, at each experimental time, the membrane was transferred into a fresh tube of 4 mL sterile DMEM, and the previous tube was stored at −20°C before ELISA quantification. The membrane transfer was done at 1 h (T0), 4 h (T1), 24 h (day 1, T2), 168 h (day 7, T3). Samples were kept at 37°C in a humified 5% CO_2_ atmosphere incubator. Final centrifugation of the 4 mL of DMEM in each tube (15,000 rpm for 10 min) was performed to remove residual particulates. Approximately 1 mL of solution was then collected and stored at −20°C before ELISA quantification.

When all the samples were collected, TGF-β1, insulin-like growth factor-1 (IGF-1), PDGF-AB, and VEGF-A were quantified with a commercially available canine ELISA kit (Cat. No. MBS2607521, MyBioSource). All the samples and ELISA kits were prepared according to the manufacturer's instructions.

### Data Analysis

Data were analysed using IBM1 SPSS1 Statistics Version 27.0 (IBM Corporation). Data for macroscopic analysis were analysed using descriptive statistics by reporting the mean and standard deviation (mean ± SD). The mean ± SD of the CBC value was calculated and compared with CBC range values in dogs.

Friedman's ANOVA test for related samples was used to assess whether a statistically significant difference was observed for each GF quantity over time intervals T0, T1, T2, and T3. Multiple comparisons were performed by the *post-hoc* Tukey-Kramer test. The significance level for all statistical tests was set a priori at *p* ≤ 0.05.

Statistical analysis of demographic variables (age, sex, and body condition score BCS) and the average of growth factors determined by the ELISA assay was performed using Prism 7.0 for Windows (GraphPad software Inc.).

Kolmogorov-Smirnov was used to assess whether the values were distributed according to a Gaussian distribution. The level of statistical significance was assessed by *t*-test or Wilcoxon-Mann-Whitney test. The results were expressed as mean ± Standard Error of the Mean (SEM).

## Results

One hundred twenty-eight dogs met the inclusion criteria; the other 172 dogs were excluded since 100 were positive to leishmaniosis (title > 1:60); 15 dogs received NSAIDs and corticosteroids for orthopaedics and dermatology issues the week before blood sampling; 15 dogs had BCS score of 2; five female dogs had mammary neoplasia (confirmed with FNA cytology); 10 dogs resulted positive for Ehrlichia canis and 10 dogs had severe CBC alterations.

The study population consisted of 18 females (14%), 25 neutered females (19.5%), 62 males (48.4%), and 23 neutered male (17.9%). The mean age was 60.47 months (±22; range 18–110 months), and the mean body weight was 31.9 kg (±5.11; range 20.5–44 kg). Table 2 shows the distribution of canine breeds. The CBC results were all in the normal range of values (Table 3).

**Table 2:** Distribution of breed and sex in study population.

**Breed**	**Sex**		**Total**
	**Male**	**Female**	
Mixed breed	46 (8 of which were neutered)	30 (16 of which were spayed)	76
Labrador retriever	5 (4 of which were neutered)		5
Pitbull terrier	3	1 (spayed)	4
Setter	1		1
TOTAL	55	31	86

**Table 3:** CBC mean values (±SD) of study population.

	**Mean value**	**SD**	**Range**
RBC	7.13 M/μL	±0.63	5.65–8.87
WBC	10.33 K/μL	±1.63	5.05–16.76
PLT	322.74 K/μL	±85.51	148–484

*SD, standard deviation; RBC, red blood cell; WBC, white blood cell; PLT, platelet*.

From 128 dogs we obtained and centrifuged two blood sample rates for a total of 256 blood samples. From these only 86 dogs gave us 2 L-PRF clots (total 172; 67.18%). Forty-two dogs (32.8%) gave us unsuitable clots and thus they were excluded from the study. From 172 L-PRF clots, we obtained 172 L-PRF membranes with a mean length of 1.97 cm (±0.89) (range: 0.5–4.0 cm), a mean width of 0.95 cm (±0.36) (range 0.5–2.0 cm), and a mean weight of 0.46 g (±0.20) (range 0.1–1.1 g).

**Figure 2:**
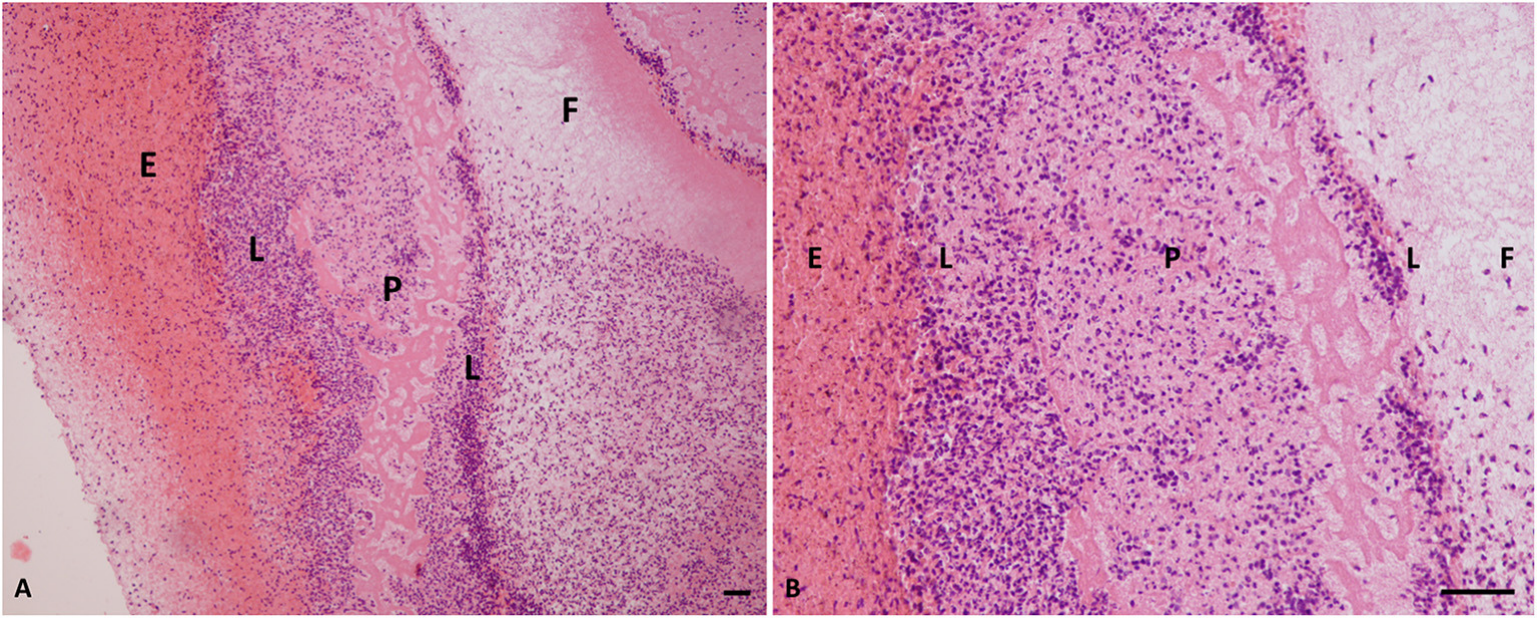
Histology of L-PRF clot. **(A)** Cellular portion of a PRF clot (×10 magnification). **(B)** PRF clot (×40 magnification). P, platelets; E, erythrocytes; F, fibrin; L, leukocytes; PRF, platelet-rich fibrin. Scale bar = 50 micron.

Histological analysis of the L-PRF membranes showed that there were five zones or layers, based on the distribution and density of blood cells in this biomaterial (Figure 2). The first layer was composed primarily of erythrocytes, followed by a transitional zone of leukocytes, erythrocytes, and platelets, a layer of leukocytes, a layer of platelets, and then a layer of fibrin. Erythrocytes were stained red, whereas platelets and platelet aggregates were stained dark pink. The fibrin network was almost white and contained few cells. Semiquantitative assessment of membrane histology showed the following results: (1) Zone layers in the membrane: a well-demarcated pattern (score 1) in 95% of the specimens; (2) Number of cell layers at border: the percentage of more than 10 cell layers (score 1) was observed in 92% of the specimens; (3) Aggregation of cells in the cell layer zone: the L-PRF membranes showed heavy aggregation (score 1) in the 90% of the specimens; (4) Cell border morphology: all the membranes (100%) showed very clear cell wall morphology.

The mean values ±SD of GFs released over time points are shown in Table 4.

**Table 4:** ELISA Test Results at Time Points T0–T3.

**Growth factor**	**Mean value ±SD**	**Mean value ±SD**	**Mean value ±SD**	**Mean value ±SD**
	**T0**	**T1**	**T2**	**T3**
VEGF (pg/mL)	539.93 ± 85.58	515.44 ± 161.1	340.18 ± 70.72	433.53 ± 42.31
PDGF-AA (ng/mL)	9.47 ± 0.95	4.04 ± 2.44	2.67 ± 1.80	4.65 ± 2.47
IGF-1 (pg/mL)	24,305.79 ± 4,255.37	15,386 ± 7,934.96	16,718.72 ± 4,018.45	16,367.35 ± 2,211.9
TGF-β1 (pg/mL)	334.40 ± 153.75	621.56 ± 245.28	507.47 ± 175.53	573.91 ± 151.59

*SD, standard deviation; VEGF, vascular endothelial growth factor; PDGF, platelet-derived growth factor; IGF-1, insulin-like growth factor-1; TGF-β1, transforming growth factor beta 1*.

The Friedman's ANOVA test results for related samples showed that there were statistically significant differences in VEGF, PDGF-AA, IGF-1 and TGF-β1 after different time intervals (*p* ≤ 0.01).

**Figure 3:**
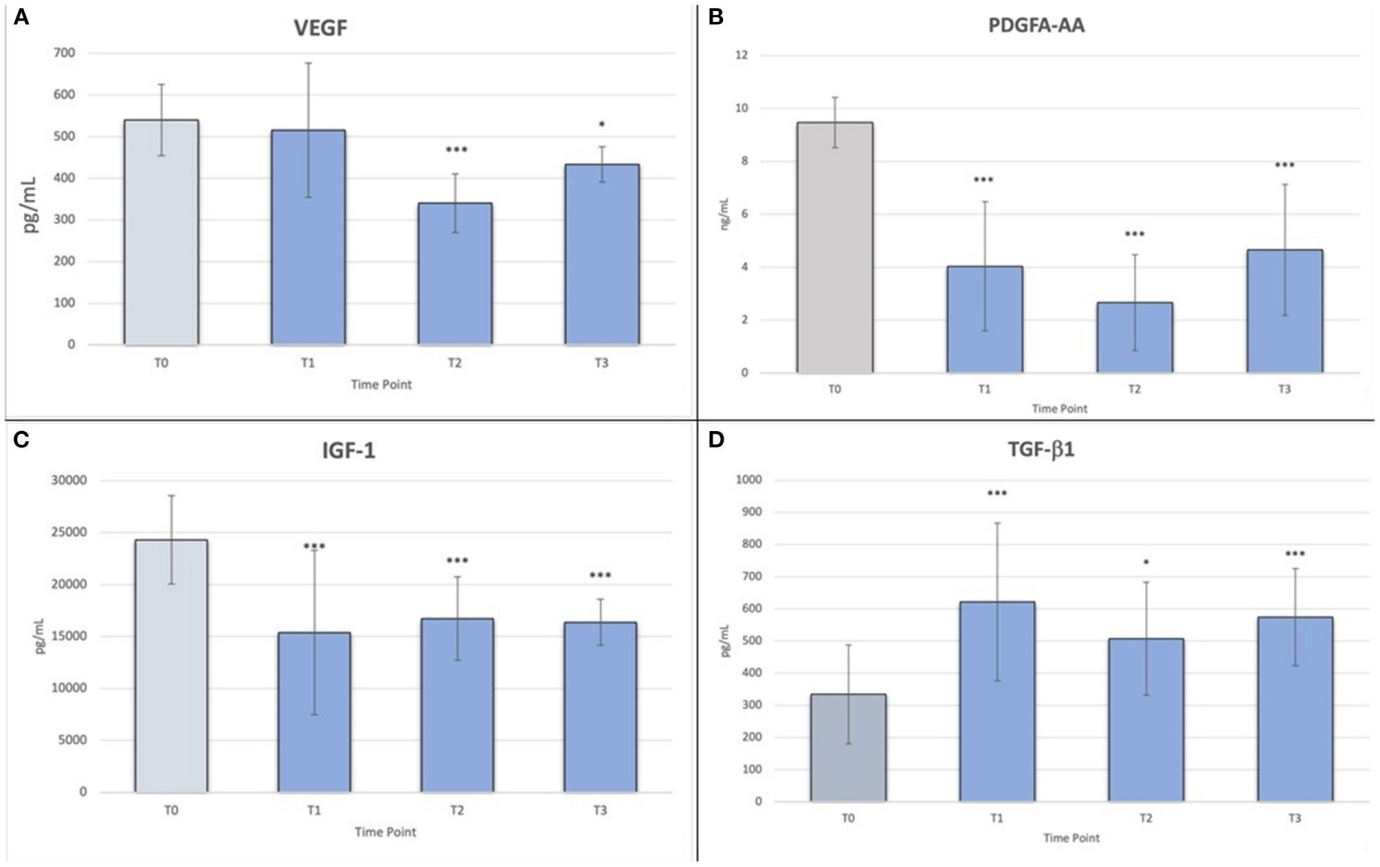
**(A)** Friedman's ANOVA test values for VEGF; *p < 0.05; ***p < 0.001; **(B)** Friedman's ANOVA test values for PDGF-AA; ***p < 0.001; **(C)** Friedman's ANOVA test values for IGF-1; ***p < 0.001; **(D)** Friedman's ANOVA test values for TGF-β1; *p < 0.05, ***p < 0.001.

The results of the multiple comparisons test (*post-hoc* Tukey-Kramer test) indicated statistically significant differences for VEGF between T0 and T2 (*p* < 0.001), T0 and T3 (*p* < 0.01), T1 and T2 (*p* < 0.001), T1 and T3 (*p* < 0.05), and T2 and T3 (*p* < 0.05) (Figure 3A). In particular, there was a decrease in VEGF concentration from T0 to T2, whereas at T3, there was a slight increase.

For PDGF-AA, there were statistically significant differences between T0 and T1, T0 and T2, and T0 and T3 (*p* < 0.001), and between T2 and T3 (*p* < 0.05) (Figure 3B). Although there was a decrease in mean concentration between T1 and T2 and a slight increase between T2 and T3, these differences were not statistically significant.

For IGF-1, similar to the previous GFs, there were statistically significant differences between T0 and T1, T0 and T2, and T0 and T3 (*p* < 0.001) (Figure 3C). The mean concentration decreased slightly over time.

Statistical analysis of demographic variables (age, sex, and body condition score BCS) and the average of growth factors determined by the ELISA assay showed a statistical significance only for age and BCS, respect to VEGF factor. In fact, it seems that a slightly higher production of VEGF is statistically associated with a young age and an optimal body weight. Results are showed in Table 5.

**Table 5:** Statistical analysis of demographic variables and mean of growth factors determined by ELISA test.

**Factor**	***n***	VEGF^*^(pg/mL)	**SEM^#^**	***P***	**PDGF-AA^§^(ng/mL)**	**SEM^#^**	***P***	**IGF-1^$^ (pg/mL)**	**SEM^#^**	**P**	**TGF-β1^¥^(pg/mL)**	**SEM^#^**	**P**
**Age**													
Young adult (1–5 years)	6	467.8	26.4		5.005	0.427		16,983	458		516.5	44.51	
				0.084			0.629			0.233			0.866
Adult (>5 years)	12	420.7	12.54		5.284	0.308		18,494	703.7		506.7	30.86	
**Gender**													
Male	11	429.8	13.6		5.28	0.403		19,035	772.1		503.6	28.1	
				0.727			0.7304			0.333			0.790
Female	9	438.8	23.2		5.10	0.207		16,980	657.1		517.6	47.7	
**BCS**													
Ideal	14	442.9	14.11		4.948	0.327		17,921	638.3		530.6	34.01	
				0.0377			0.1723			0.534			0.270
Slightly overweight	8	417.1	22.65		5.664	0.342		18,673	1,114		472.1	32.8	
Total	20	438.81											

* *VEGF, vascular endothelial growth factor, mean value*.

§ *PDGF, platelet-derived growth factor, mean value*.

$ *IGF-1, insulin-like growth factor-1, mean value*.

¥ *TGF-β1, transforming growth factor beta 1, mean value*.

# *SEM Standard Error of the Mean*.

Finally, for TGF-β1, there was a statistically significant difference only between T0 and T1, T0 and T3 (*p* < 0.001), and T0 and T2 (*p* < 0.05) (Figure 3D).

## Discussion

This prospective study aimed to standardise L-PRF clot production in dogs based on a centrifugation protocol proposed by Crisci et al. in horses (24). We obtained L-PRF membranes using an L-PRF Wound Box and performed macroscopic and histological evaluations and quantitative *in vitro* analysis of GFs over 7 days.

The PRF production technique is easy, cheap, does not require specialised equipment, and produces consistent clots. However, some drugs (i.e., NSAIDs or corticosteroids) can inhibit primary hemostasis via the inhibition of cyclooxygenase-1, leading to decreased thromboxane-A2 synthesis and reduced platelet aggregation (20–23). In our study population, the sampling procedure was well-tolerated, and the CBC values were within the physiological range in all dogs enrolled. Moreover, the inclusion criteria allowed the selection of a study population in which no one medication or disease could alter the normal clotting process or the platelet count. The success in producing L-PRF clots depends entirely on the speed of blood collection and transfer to the centrifuge because the PRF clot is formed by natural polymerization during centrifugation (26). The time between sampling and centrifugation is important in clot formation. An interval longer than 2 min yields an amorphous blood clot, making the compression difficult, or yields an unsuitable membrane (18). Crisci et al. reported that the best results in horses were obtained with an interval of <2 min between blood withdrawal and centrifugation, and with centrifugation at temperatures in the range of 21–30°C (24). Our data are consistent with the current literature. Indeed, in our sample, 42/128 clots (32.8%) were unsuitable. In all these cases, the delay between collection and centrifugation was over 2 min, and room temperature exceeded 30°C. However, 86/128 clots (67.18%) were suitable when the room temperature was 25°C and the delay was not over 2 min. These data confirmed our first hypothesis about the effectiveness of protocol used to obtain homogeneous L-PRF clots in dogs. In our experience, the homogeneity of the clots is related to the detail of the centrifugation protocol (24). Indeed, the initial soft spin at 2 min at 2,700 rpm and 4 min at 2,400 rpm separates the plasma and platelets from the red cells and partially from the white cells (27). The second hard spin for 3 min at 3,000 rpm condenses the fibrin further and produces a rich fibrin structure characterised by a dense fibrin clot.

To the best of our knowledge, no previous study has standardised L-PRF membrane production in dogs. McLellan and Plevin (28) and Crisci (29) showed that equine PRF is similar to human PRF, providing an immediate and constant source of tissue GFs. Soares et al. (10) demonstrated that feline and canine PRFs could be obtained from a small volume of blood in four dogs and four cats, and they reported the GF release profile of the clots. In surgery, three-dimensional autologous scaffolds that can be sutured on a lesion are widely used. The L-PRF membranes prepared by clot squeezing with gauze have been used principally in alveolar-dental lesions (15–17). The membranes produced by constant pressure, such as those produced by the L-PRF Wound Box, are different to those produced by gauze squeezing. Crisci et al. standardised the use of the L-PRF Wound Box to produce L-PRF membrane in horses (24). The L-PRF Wound Box is commercially available in a variety of shapes and pressure plate weights. The thickness, width, and length of the membrane are directly related to compression time and the pressure plate weight. The pressure plate weight and compression time are not clearly defined in the available literature in dogs. In the present study, we used an L-PRF Wound Box with a pressure plate of 148 g to apply a constant pressure of 142.437 Pa/cm^2^ for 15 min. The mean size of membranes obtained was 2 × 2 cm, making them suitable for clinical application. These data are consistent with our second hypothesis, confirming the effectiveness of the L-PRF Wound Box (24) for producing membranes ideal for clinical usage in dogs. Membrane lengths were 0.5–4 cm and widths were 0.5–2 cm. According to our experience, the difference in sizes is related to the time interval between centrifugation and clot compression. Castro et al. (18) identified this time interval as crucial because the longer the time interval, the smaller the membrane (18). Our results were affected by logistical difficulties in the early stages of the study, and the increased time between centrifugation and clot compression reduced the membrane size. Improving our coordination and timing enabled us to overcome this problem; subsequently, we obtained larger membranes.

Regardless of the membrane dimensions, the histological examination revealed a specific histoarchitecture, characterised by five layers consisting of the platelets, leukocytes, and red blood cells trapped in a fibrin mesh network. This was because during the synthesis of autologous fibrin scaffolds, the three-dimensional fibrin nanoscaffold incorporated the platelets in a non-diffusible mode and bound platelet- and plasma-derived GFs before they attached to their corresponding cell-surface receptors. This histoarchitecture is also described in the human and horse models (24, 30, 31). These results fulfil the evaluation required to standardise L-PRF production in dogs.

In humans, PRF releases GFs consistently over time, whereas PRP releases a high level of GFs during the first 24 h (6, 32). In particular, PRF released more GFs at later time points and contained more GFs overall in the fibrin matrix. We used a specific ELISA kit to evaluate and quantify the GFs released by L-PRF membranes. Our results were consistent with those from human and veterinary medicine (6, 10). For VEGF, PDGA-A, and IGF-1, we detected an initial percentage decrease and a moderate increase between 24 and 168 h. In contrast, the results for TGF-β1 were different from other studies (10); there was an increase of the content of this GF over time controls. These trends could be explained by the living cells (leukocytes and platelets) in L-PRF that contribute to the slight increase in total GF accumulation after 10 days (32). Finally, in human medicine it has been described a correlation between plasma level of VEGF with the age in particular it was statistically higher in people older than 40 years (33); further experimental investigations are needed to estimate the relationship between VEGF and age and weight in veterinary medicine.

The results of our study confirm that the appropriate centrifugation of canine venous blood quickly harvested, stored, and processed produces a dense, well-organised L-PRF fibrin mesh. Our assessment of the GF release over time confirms the potential of this bioscaffold to enhance, regulate, and promote direct stem cell migration and induction of healing pathways. The standardisation of a production protocol for L-PRF clots and membranes in dogs and the assessment of macroscopic and histological features is required to evaluate the clinical effectiveness of L-PRF membranes objectively.

However, further *in vivo* studies are necessary to evaluate the potential clinical applications of L-PRF membranes with these microscopic and macroscopic characteristics, especially for treating wounds with tissue loss in which the healing process appears to be delayed or in fistulas, and in all cases in which the enhancement and regulation of the healing process are needed.

## Data Availability Statement

The original contributions presented in the study are included in the article/supplementary material, further inquiries can be directed to the corresponding author/s.

## Ethics Statement

The animal study was reviewed and approved by Ethical Animal Care and Use Committee of the University of Naples Federico II (prot. No. PG/2018/0113033 of 28/11/2018).

## Author Contributions

GD and GFa performed experimental design. CC, FA, and GD performed the sampling and macroscopic evaluations. DD performed histological evaluations of the membranes L-PRF. GFe performed the ELISA. CC performed the data analysis and edited the paper. CC and GD interpreted the results and wrote the manuscript. FL supervised and reviewed the paper. GFa approved the paper. All authors read and approved the final version of the paper.

## Funding

GD received grant from P.R.I.N. 2017 prot. N. 2017F8ZB89_003. ERC:LS7. The funders had no role in study design, data collection and analysis, decision to publish, or preparation of the manuscript.

## Conflict of Interest

The authors declare that the research was conducted in the absence of any commercial or financial relationships that could be construed as a potential conflict of interest.

## Publisher's Note

All claims expressed in this article are solely those of the authors and do not necessarily represent those of their affiliated organizations, or those of the publisher, the editors and the reviewers. Any product that may be evaluated in this article, or claim that may be made by its manufacturer, is not guaranteed or endorsed by the publisher.
